# Multimodal Bone Fragility Profiling in People Living with HIV: Trabecular Bone Score, Calcaneal Quantitative Ultrasound, and Sarcopenia Screening

**DOI:** 10.3390/medicina62030603

**Published:** 2026-03-23

**Authors:** David Vladut Razvan, Jenel Marian Patrascu, Ovidiu Rosca, Iulia Georgiana Bogdan, Livia Stanga, Adrian Vlad, Camelia Vidita Gurban

**Affiliations:** 1 Doctoral School, Faculty of Medicine, Victor Babes University of Medicine and Pharmacy, 300041 Timisoara, Romania; vladut-razvan.david@umft.ro; 2 Centre for Molecular Research in Nephrology and Vascular Disease, Faculty of Medicine, Victor Babes University of Medicine and Pharmacy, 300041 Timisoara, Romania; 3 Orthopaedics II Research Center, “Pius Brinzeu” Emergency County Hospital, 300723 Timisoara, Romania; jenel.patrascu@umft.ro; 4 Department of Orthopedics and Traumatology, Victor Babes University of Medicine and Pharmacy, Efimie Murgu Square 2, 300041 Timisoara, Romania; 5 Methodological and Infectious Diseases Research Center, Department of Infectious Diseases, Victor Babes University of Medicine and Pharmacy, 300041 Timisoara, Romania; ovidiu.rosca@umft.ro (O.R.); iulia-georgiana.bogdan@umft.ro (I.G.B.); 6 Discipline of Microbiology, Victor Babes University of Medicine and Pharmacy, 300041 Timisoara, Romania; 7 Department of Internal Medicine II, Division of Diabetes, Nutrition and Metabolic Diseases, Victor Babes University of Medicine and Pharmacy, 300041 Timisoara, Romania; 8 Department IV Biochemistry and Pharmacology, Discipline of Biochemistry, Victor Babes University of Medicine and Pharmacy, 300041 Timisoara, Romania; gurban.camelia@umft.ro

**Keywords:** HIV infections, osteoporosis, bone density, sarcopenia, tenofovir

## Abstract

*Background and Objectives:* Bone fragility in people living with HIV (PLWH) reflects both reduced bone mineral density (BMD) and impaired microarchitecture, while functional decline may further amplify fracture vulnerability. This study evaluated whether adding a pragmatic sarcopenia screen improves bone fragility characterization beyond DXA-BMD, trabecular bone score (TBS), calcaneal quantitative ultrasound (QUS), and biomarkers, and explored the relationship between tenofovir disoproxil fumarate (TDF) exposure and microarchitectural impairment. *Materials and Methods:* In this single-center cross-sectional study at Victor Babeș University of Medicine and Pharmacy Timișoara, 98 adults on stable ART underwent DXA (T-scores), lumbar TBS (reported as TBS × 100), calcaneal QUS (SOS/BUA), and bone turnover markers (CTX, P1NP, 25(OH)D). Sarcopenia screening used handgrip strength and 4 m gait speed. Associations were tested using group comparisons, correlations, and multivariable modeling for degraded TBS (TBS × 100 < 124.0). *Results:* Sarcopenia screen-positive participants (*n* = 28) had lower TBS (123.8 vs. 127.7, *p* = 0.02), lower lumbar T-score (−1.7 vs. −1.2, *p* = 0.014), lower SOS (1523.3 vs. 1548.8 m/s, *p* = 0.002), and higher CTX (0.6 vs. 0.4 ng/mL, *p* < 0.001), with less frequent viral suppression (60.7% vs. 85.7%, *p* = 0.006). With >5 years TDF exposure (*n* = 28), degraded TBS prevalence was 82.1% vs. 40.0% in never-exposed (*p* = 0.001), alongside lower TBS (123.1 vs. 129.8, *p* < 0.001) and higher CTX (0.6 vs. 0.4 ng/mL, *p* < 0.001). Viral suppression independently reduced odds of degraded TBS (aOR 0.3, 95% CI 0.1–0.9; *p* = 0.034). *Conclusions:* In PLWH, prolonged TDF exposure and functional impairment co-occur with worse densitometric and microarchitectural profiles; viral suppression shows an independent protective association with microarchitecture.

## 1. Introduction

Bone disease has emerged as a clinically relevant non-communicable comorbidity in people living with HIV (PLWH), largely because contemporary antiretroviral therapy (ART) has transformed HIV into a chronic condition with prolonged survival. In this setting, osteopenia, osteoporosis, and morphometric vertebral fractures are encountered earlier than expected, reflecting an overlap between traditional risk factors (smoking, low BMI, hypogonadism, vitamin D deficiency), HIV-related immune–inflammatory perturbations, and cumulative ART exposure across decades of care [1,2,3,4]. Current expert guidance therefore frames skeletal health as part of routine comprehensive HIV management, not an “optional” add-on [5].

A key clinical nuance is that bone loss in HIV is not purely a late complication: a reproducible drop in bone mineral density (BMD) occurs soon after ART initiation, with trials and cohorts reporting roughly a 2–6% decline over the first 24–48 weeks, followed by relative stabilization [2]. This early “reset” in skeletal homeostasis is clinically meaningful because it may move borderline patients into higher-risk zones, particularly when compounded by tenofovir disoproxil fumarate-associated renal–phosphate effects or other regimen- and host-related factors that accelerate bone turnover [2,3]. These dynamics motivate pragmatic risk stratification strategies that go beyond a single densitometric snapshot.

International recommendations emphasize targeted screening rather than universal DXA, typically prioritizing individuals with older age, prior fragility fracture, chronic glucocorticoid exposure, high falls risk, or other major fracture predictors [5]. In Europe, the latest major update of the European AIDS Clinical Society (EACS) guidelines similarly reinforces structured comorbidity assessment, including bone health, within routine HIV care pathways, which is particularly relevant for real-world clinics balancing competing metabolic and cardiovascular priorities [6]. However, even with guideline-driven screening, the operational problem remains: fracture risk in HIV is frequently multifactorial, and purely “bone-only” algorithms may under-capture functional contributors to fragility.

Dual-energy X-ray absorptiometry (DXA) remains the cornerstone for BMD measurement, yet it quantifies areal density and cannot directly capture trabecular microarchitecture or bone material properties, domains that may be disproportionately affected by inflammation, endocrine disruption, and ART-related remodeling [5]. Trabecular bone score (TBS), derived from lumbar DXA images, offers a practical surrogate for microarchitectural integrity without additional radiation. Importantly, in PLWH, impaired TBS has been linked to subclinical vertebral fractures and may provide incremental information even when BMD is not frankly osteoporotic, supporting the concept of a “bone quality gap” in HIV-related fragility phenotypes [7,8].

Access constraints also matter. In resource-limited environments, such as parts of Romania, DXA availability and throughput may be limited, creating demand for scalable adjuncts. Calcaneal quantitative ultrasound (QUS) is attractive because it is portable and radiation-free, and HIV cohorts have shown clinically relevant correlations between QUS indices (e.g., stiffness) and DXA-derived BMD, supporting QUS as a screening or triage tool when densitometry access is constrained [9,10]. Recent work in PLWH also highlights the value of combining DXA-derived metrics (BMD, TBS) with QUS and bone turnover markers to improve morphometric vertebral fracture profiling, reinforcing a multimodal approach that remains feasible outside highly resourced centers [11].

Even a refined skeletal assessment can still miss a broader fragility phenotype driven by falls propensity and functional reserve. Sarcopenia, conceptualized as impaired muscle strength and/or performance with clinically relevant outcome links, has evidence-based operational definitions emphasizing grip strength and gait speed because these measures best discriminate adverse outcomes [12]. In HIV-relevant populations, validated cut-points for weakness and slowness have been tested in men and women with or at risk for HIV infection, demonstrating the practicality of incorporating simple bedside performance metrics into risk stratification pathways [13]. Furthermore, systematic syntheses suggest that sarcopenia is not rare in PLWH and is associated with adverse clinical trajectories, while in the general older-adult literature sarcopenia is consistently associated with falls and fractures, providing a mechanistic and epidemiologic rationale for integrating muscle function into fragility profiling [14,15]. Therefore, this cross-sectional study in a Romanian tertiary HIV center evaluates whether adding a feasible sarcopenia screening layer improves bone fragility characterization beyond DXA, TBS, QUS, and routine biomarkers; it is hypothesized that sarcopenia-positive individuals will exhibit worse densitometric and microarchitectural profiles and that TBS impairment will be only partially explained by BMD categories.

## 2. Materials and Methods

### 2.1. Study Design & Setting

This was a single-center, cross-sectional observational study conducted in the HIV outpatient services and affiliated imaging/laboratory units at Victor Babeș University of Medicine and Pharmacy Timișoara. The Timișoara HIV outpatient service functions as one of the principal regional referral centers for HIV care in western Romania, managing approximately 900–1000 adults living with HIV, and its patient demographic—including both perinatally and horizontally infected individuals across a wide age range—broadly reflects the epidemiologic profile of PLWH in the region, although referral enrichment toward more complex cases is possible given its tertiary, university-affiliated status. The design prioritized feasibility within a resource-limited Romanian context: all assessments were performed during routine care visits or a closely aligned “one-stop” evaluation pathway.

Data collection was planned over an approximate 18–24-month window to allow consecutive recruitment without disrupting clinical workflows. The protocol was designed to be non-interventional: imaging and laboratory tests were part of standard metabolic and comorbidity assessment, while sarcopenia screening used low-cost bedside tools (handgrip dynamometer and timed walk).

### 2.2. Participants and Eligibility Criteria

Adults (≥18 years) with confirmed HIV infection were eligible if they were receiving stable combination ART for at least 12 months and were referred for bone health evaluation (age threshold, clinician concern, prior fracture history, prolonged corticosteroid exposure, or other standard indications). All participants were required to provide written informed consent and be able to complete the functional testing protocol.

Exclusion criteria were selected to limit major confounding of bone assessment: pregnancy, active malignancy with known bone involvement, advanced chronic kidney disease (stage 4–5), primary metabolic bone disease (including uncontrolled hyperparathyroidism and severe renal osteodystrophy), or current high-dose systemic glucocorticoids. current or recent prolonged supraphysiological systemic glucocorticoid therapy, recent orthopedic surgery affecting DXA or QUS assessment sites (within 12 months), and current use of bone-active pharmacotherapy other than vitamin D supplementation. Menopausal status was recorded but was not an exclusion criterion, as postmenopausal women represent a clinically relevant subgroup in HIV bone health assessment. Patients with acute severe illness at assessment were deferred. The final analytic sample was 98 participants.

### 2.3. Bone, Laboratory, and Sarcopenia Assessments

DXA measured lumbar spine (L1–L4) and proximal femur BMD, recording site-specific T-scores. BMD categories followed WHO thresholds using the lowest site T-score: normal (≥−1.0), osteopenia (−1.0 to −2.5), and osteoporosis (≤−2.5). TBS was computed from lumbar DXA images using dedicated software; to keep reporting consistent with one-decimal tables, TBS was presented as TBS × 100 (e.g., 127.6 corresponds to TBS 1.276). “Degraded” TBS was defined as TBS × 100 < 124.0, partially degraded 124.0–130.0, and normal >130.0.

Calcaneal QUS was performed on the non-dominant heel with standardized operator procedures and device quality checks. Parameters included SOS (m/s) and BUA (dB/MHz). Fasting blood tests included serum CTX and P1NP (reference turnover markers) and 25(OH) vitamin D.

A pragmatic sarcopenia screen was implemented to match resource constraints. Handgrip strength was measured with a dynamometer using standardized posture and best-of-three attempts; low grip was defined by sex-specific cutoffs consistent with common European practice. Gait speed was measured over 4 m at usual pace; slow gait was defined as <0.8 m/s. Participants were categorized as sarcopenia screen-positive if they met low grip strength and/or slow gait speed criteria.

### 2.4. Statistical Analysis

Continuous variables were summarized as mean ± SD; categorical variables as n (%). Normality was assessed by visual inspection and Shapiro–Wilk testing. Two-group comparisons (sarcopenia-positive vs. negative) used independent-samples *t*-tests for approximately normal variables and Mann–Whitney U tests for non-normal distributions. Three-group comparisons (TDF exposure strata) used one-way ANOVA with post hoc testing where appropriate; categorical outcomes used chi-square tests (or Fisher’s exact test when expected counts were small).

Pearson correlation coefficients quantified linear associations between TBS × 100 and relevant clinical/biochemical variables. A multivariable logistic regression model examined predictors of degraded TBS (TBS × 100 < 124.0), reporting adjusted odds ratios (aOR) with 95% confidence intervals. Model predictors were chosen a priori based on feasibility and clinical plausibility (age, BMI, HIV duration, nadir CD4+ category, viral suppression, cumulative TDF exposure category, sarcopenia status, CTX). Statistical significance was set at two-sided *p* < 0.05.

## 3. Results

Patients who screened positive for sarcopenia were notably older (53.4 vs. 42.8 years, *p* < 0.001) and had a longer HIV duration (12.6 vs. 10.3 years, *p* = 0.03), with evidence of more advanced historical immunosuppression (lower nadir CD4+: 206.3 vs. 258.7 cells/mm^3^, *p* = 0.029). Viral suppression was less frequent in the sarcopenia group (60.7% vs. 85.7%, *p* = 0.006). Bone and muscle-related measures showed the clearest separation: CTX was higher (0.6 vs. 0.4 ng/mL, *p* < 0.001), grip strength was substantially lower (23.1 vs. 33.7 kg, *p* < 0.001), and gait speed was slower (0.7 vs. 1.1 m/s, *p* < 0.001). Skeletal quality also appeared worse in those with sarcopenia, with a lower lumbar spine T-score (−1.7 vs. −1.2, *p* = 0.014), lower TBS (123.8 vs. 127.7, *p* = 0.02), and reduced calcaneal SOS (1523.3 vs. 1548.8 m/s, *p* = 0.002) (Table 1).

Longer TDF exposure showed a clear dose–response pattern toward worse bone density, weaker microarchitecture, and higher bone turnover. Compared with never-exposed patients, those with >5 years of TDF were older (49.3 vs. 42.3 years, *p* = 0.004) and had substantially lower DXA T-scores across all sites—lumbar spine (−1.7 vs. −0.9), total hip (−1.3 vs. −0.6), and femoral neck (−1.6 vs. −0.9), all *p* < 0.001. TBS declined stepwise (123.1 vs. 129.8, *p* < 0.001) and calcaneal SOS also decreased (1524.2 vs. 1557.7 m/s, *p* < 0.001), with BUA lower as exposure increased (104.8 vs. 111.6 dB/MHz, *p* = 0.034). Bone turnover markers were higher with prolonged exposure: CTX rose (0.6 vs. 0.4 ng/mL, *p* < 0.001) and P1NP increased (60.0 vs. 48.8 µg/L, *p* = 0.02). Clinically, the proportion with degraded TBS (<124.0) was much higher in the >5-year group (82.1%) than in never-exposed patients (40.0%), *p* = 0.001 (Table 2).

This table shows that “bone quality” (TBS) worsens as BMD category declines, and the distribution of TBS categories differs significantly across BMD groups (chi-square *p* = 0.006). Even among patients with normal BMD, over one-quarter had degraded TBS (27.0%), suggesting microarchitectural impairment can exist despite normal density. In osteopenia, degraded TBS was common (38.6%), and in osteoporosis it was the dominant pattern (58.8%). Vertebral fracture prevalence increased numerically from normal BMD (8.1%) to osteopenia (18.2%) and osteoporosis (23.5%), but this trend did not reach statistical significance (*p* = 0.236), consistent with limited power for fracture comparisons in the smallest subgroup (Table 3).

TBS correlated most strongly with classic skeletal measures and functional performance. Higher TBS was moderately-to-strongly associated with higher lumbar spine T-score (r = 0.6, *p* < 0.001) and total hip T-score (r = 0.5, *p* < 0.001), supporting that better density aligns with better microarchitecture. TBS also correlated positively with calcaneal SOS (r = 0.4, *p* < 0.001) and with grip strength and gait speed (both r = 0.4, *p* < 0.001), linking microarchitecture to overall musculoskeletal health. In contrast, older age (r = −0.4, *p* < 0.001), longer HIV duration (r = −0.3, *p* = 0.002), and higher CTX (r = −0.3, *p* = 0.001) were associated with lower TBS. Vitamin D showed a small but statistically significant positive correlation (r = 0.2, *p* = 0.045) (Table 4).

After adjusting for multiple factors, the only predictor that remained statistically significant was viral suppression, which was associated with substantially lower odds of degraded TBS (aOR 0.3, 95% CI 0.1–0.9, *p* = 0.034). Other variables showed trends but did not meet significance—age (aOR 1.4 per 10 years, *p* = 0.106) and BMI (aOR 0.9 per 1 kg/m^2^, *p* = 0.072) suggested possible directionality, but uncertainty remained. Notably, prolonged TDF exposure (>5 years) and sarcopenia were not independently significant in this multivariable model (both *p* > 0.3–0.5), implying that their effects on TBS may overlap with other variables or be clearer in stratified/non-linear analyses rather than a single binary endpoint model (Table 5).

Adding DXA T-score information improved fracture discrimination more than adding TBS or sarcopenia in this dataset. The clinical-only model (A) had an AUC of 68.3, which increased to 72.5 after adding the lowest T-score (Model B; ΔAUC + 4.2), although this improvement was borderline (LRT *p* = 0.081). Adding TBS (Model C) produced only a small further gain (AUC 74.0; ΔAUC +1.5; *p* = 0.484), and adding sarcopenia (Model D) again increased AUC modestly (75.6; ΔAUC + 1.6; *p* = 0.377), without statistically significant incremental improvement (Table 6).

This analysis suggests that some factors affect TBS more clearly in the overall “average” range and in the lower (more vulnerable) tail. Cumulative TDF exposure was consistently associated with lower TBS both in the mean model (β − 0.5 per year, *p* = 0.018) and at the 25th percentile (β − 0.6, *p* = 0.023), indicating a robust relationship that persists even among patients already in the lower TBS range. Age also showed a significant negative association in both approaches (mean β − 0.2, *p* = 0.013; lower-tail β − 0.3, *p* = 0.034). In contrast, probable sarcopenia, CTX, vitamin D, BMI, and viral suppression were not significant in either model (all *p* > 0.2), suggesting their relationships with TBS may be weaker, non-linear, or mediated through other pathways in this dataset (Table 7).

After controlling for key confounders (age, BMI, and HIV duration), the strongest remaining relationships centered on the agreement between microarchitecture, density, and ultrasound-based bone quality. TBS had a strong positive partial correlation with calcaneal SOS (partial r = 61.1 × 100, *p* < 0.001, q < 0.001), and also remained significantly linked to lumbar spine T-score (35.9 × 100, q = 0.002). Lumbar spine T-score was also significantly associated with calcaneal SOS (38.2 × 100, q = 0.001). Most other connections did not survive multiple-testing correction (q-values generally > 0.2) (Table 8).

**Figure 1:**
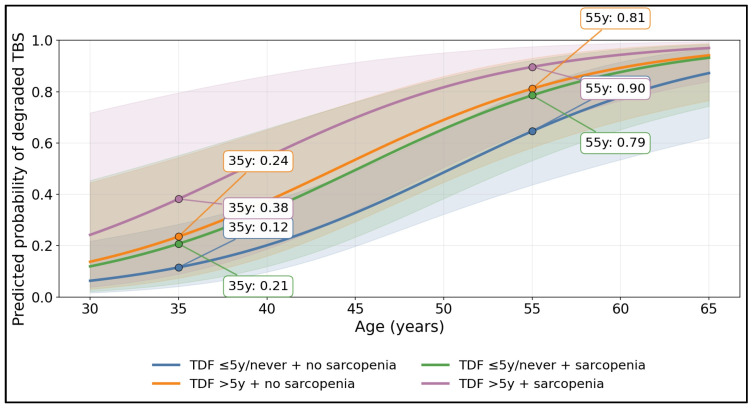
Age-, TDF-, and Sarcopenia-Stratified Predicted Probability of Degraded Trabecular Microarchitecture.

Figure 1 shows a clear age-gradient in microarchitectural deterioration, with the predicted probability of degraded TBS rising across all strata and separating meaningfully by TDF > 5 years and sarcopenia. At 35 years, predicted risk was lowest in the reference profile (TDF ≤ 5y/never + no sarcopenia) at 0.12, compared with 0.24 in TDF > 5y without sarcopenia, 0.21 in sarcopenia without prolonged TDF, and highest in combined exposure (TDF > 5y + sarcopenia) at 0.38. By 55 years, probabilities increased substantially and the between-group divergence persisted, reaching 0.65 (TDF ≤ 5y/never + no sarcopenia), 0.81 (TDF > 5y + no sarcopenia), 0.79 (TDF ≤ 5y/never + sarcopenia), and 0.90 (TDF > 5y + sarcopenia).

**Figure 2:**
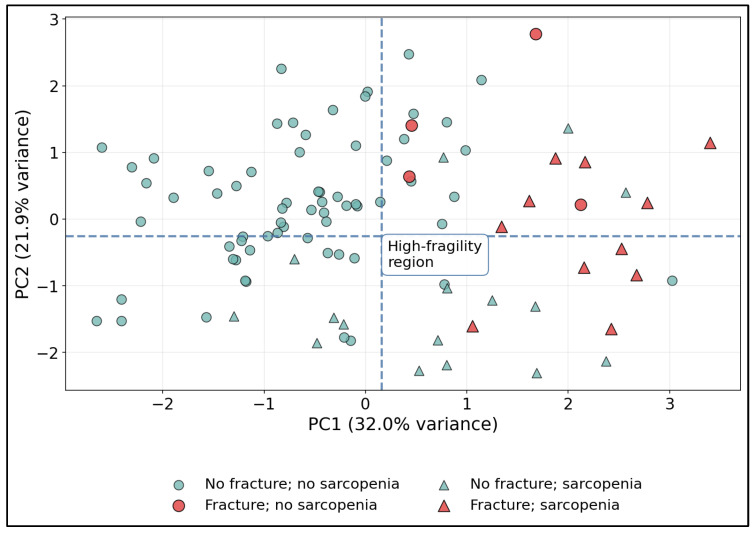
PCA-Derived Skeletal–Functional Phenotype Space and a High-Fragility Region by Fracture Status and Sarcopenia.

Figure 2 indicates that fracture risk aligns with a multidimensional pattern (not a single parameter), and that combining skeletal and functional features yields recognizable clusters that may help identify higher-risk patients even when any one measurement alone appears borderline.

**Figure 3:**
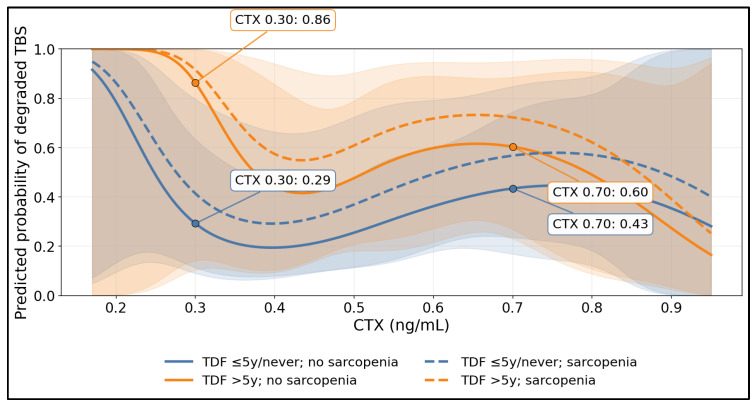
Non-Linear Relationship Between CTX and Degraded TBS Probability: Effect Modification by Prolonged TDF Exposure and Sarcopenia.

Figure 3 demonstrates a non-linear (spline) association between CTX and the predicted probability of degraded TBS, with the CTX–risk curve shifted upward by TDF > 5 years and further accentuated in sarcopenia (dashed lines). At clinically interpretable CTX points, prolonged TDF exposure markedly increased predicted risk in the non-sarcopenic stratum: at CTX = 0.30 ng/mL, the predicted probability was 0.29 for TDF ≤ 5y/never versus 0.86 for TDF > 5y, and at CTX = 0.70 ng/mL, the probability was 0.43 versus 0.60, respectively. The wide confidence bands at the extremes reflect fewer observations in tails, but the consistent upward displacement of the TDF > 5y curves supports effect modification, implying that for similar turnover levels, individuals with prolonged TDF exposure (especially with sarcopenia) may experience disproportionate microarchitectural vulnerability.

## 4. Discussion

### 4.1. Analysis of Findings

The present study adds a pragmatic “real-world” layer to HIV bone assessment by showing that prolonged tenofovir disoproxil fumarate exposure is associated not only with lower BMD but also with worse microarchitectural surrogates (TBS) and peripheral skeletal indices (calcaneal QUS), alongside higher turnover. The stepwise decline in TBS and worsening DXA T-scores across increasing TDF duration strata supports a cumulative-exposure signal and aligns with randomized switch evidence in which replacing TDF with tenofovir alafenamide (TAF) generally improves or stabilizes skeletal outcomes while maintaining virologic suppression. These patterns have been demonstrated in large switch trials of TAF coformulations, reinforcing the biological plausibility that longer TDF exposure contributes to a broader fragility phenotype beyond densitometry alone [16,17]. Notably, the co-occurrence of sarcopenia and prolonged TDF exposure in our cohort suggests a compounded fragility phenotype, as sarcopenia-positive participants demonstrated significantly higher bone resorption (CTX 0.6 vs. 0.4 ng/mL, *p* < 0.001) and worse microarchitectural indices (TBS 123.8 vs. 127.7, *p* = 0.02) alongside lower calcaneal SOS (1523.3 vs. 1548.8 m/s, *p* = 0.002), indicating that musculoskeletal functional decline and TDF-mediated skeletal toxicity may act through overlapping pathways—accelerated turnover, impaired mineralization, and reduced mechanical loading—to amplify bone damage beyond what either factor produces in isolation. Importantly, while the detrimental effect of TDF on areal bone mineral density is well established, the present study extends this evidence by demonstrating that prolonged TDF exposure is independently associated with impaired trabecular microarchitecture (TBS) and peripheral ultrasound indices (calcaneal QUS), revealing a bone quality dimension that is not captured by conventional densitometry alone and that may explain fracture risk in patients with non-osteoporotic BMD.

The biomarker profile further supports a “high-turnover, low-quality” phenotype in prolonged TDF exposure and in sarcopenia-screen–positive participants. In randomized data, switching from TDF to TAF in older, virologically suppressed adults has been associated with more favorable bone trajectories than remaining on TDF, suggesting that skeletal vulnerability may be modifiable even later in life, when competing comorbidities are common [18]. Comparable signals have also been reported in sex- and life-stage-specific populations, including women, supporting generalizability across clinically relevant subgroups [19]. Mechanistically, turnover-marker changes appear to be part of this benefit; randomized switch evidence has shown larger reductions in resorption markers after TDF-to-TAF substitution compared with continued TDF, consistent with the present cohort’s observation that CTX tracked microarchitectural risk patterns and may help contextualize disproportionate TBS impairment [20]. It should also be noted that boosted protease inhibitor (PI)-based regimens, which were used by a subset of patients in this cohort, may independently contribute to bone loss through effects on vitamin D metabolism, renal tubular function, and osteoclast activation, and could interact with TDF-related skeletal toxicity in ways that our study design did not allow us to disentangle.

A key contribution of this study is the demonstration of BMD–TBS discordance: more than one-quarter of individuals with normal BMD still exhibited degraded TBS, implying microarchitectural impairment despite preserved areal density. This “bone quality gap” has been described in other HIV cohorts using TBS, where HIV-related and cardiometabolic factors were associated with lower TBS independent of BMD, supporting the concept that microarchitecture can be adversely affected even when BMD does not meet osteoporosis thresholds [21]. Longitudinal evidence also suggests that TBS can worsen over time and may capture risk domains not fully reflected by BMD trajectories [22]. In addition, microarchitectural deficits have been demonstrated in women living with HIV, further supporting that TBS-relevant vulnerability is not restricted to a single demographic group and may relate to multifactorial host and treatment effects [23].

Although vertebral fracture prevalence increased numerically across worsening BMD categories, sequential discrimination models in this modest cross-sectional cohort showed only incremental AUC gains when adding TBS and sarcopenia to clinical factors and the lowest T-score. Limited incremental AUC is compatible with low event counts, cross-sectional timing relative to exposure, and the multifactorial nature of fragility in HIV, rather than negating clinical utility. This interpretation is supported by external evidence that standard fracture prediction tools may underperform in HIV; in women with HIV, FRAX has been shown to underestimate fracture risk, indicating calibration limitations when HIV-specific risk biology and functional contributors are incompletely represented [24]. Accordingly, the current PCA-derived phenotype space and BMD–TBS discordance can be viewed as hypothesis-generating support for integrated profiling (density + microarchitecture + peripheral screening + function + turnover) to better approximate fragility risk than any single measure.

Finally, the finding that viral suppression was independently associated with lower odds of degraded TBS supports an immune–inflammatory contribution to skeletal microarchitecture. Clinical evidence links immunosuppression history to fragility outcomes; lower CD4 counts have been associated with increased fragility fracture risk in people with HIV [25]. Mechanistic syntheses also emphasize that chronic immune activation and inflammation can alter bone remodeling and compromise bone strength, providing a plausible pathway through which incomplete suppression or intermittent viremia could contribute to microarchitectural deterioration even after accounting for conventional risk factors [26]. Taken together, these data support a practical implication for routine HIV care pathways: sustained viral suppression and ART optimization (including consideration of TDF-to-TAF switching where appropriate) should be paired with feasible functional screening and selective TBS/QUS use to identify patients who may be “high fragility” despite non-osteoporotic BMD.

These findings support a pragmatic, multimodal fragility pathway in PLWH: (i) routine attention to microarchitecture can identify a bone quality gap, including degraded TBS in a meaningful subset even when BMD is not frankly osteoporotic; (ii) calcaneal QUS may serve as a scalable adjunct for triage where DXA throughput is limited; (iii) brief functional testing (grip strength and gait speed) can flag a higher-vulnerability phenotype with higher turnover and poorer skeletal measures. Clinically, patients with prolonged TDF exposure (>5 years) warrant intensified skeletal surveillance and risk mitigation, while durable viral suppression may confer measurable microarchitectural benefit for degraded TBS. In everyday clinical practice, these findings underscore three actionable implications: (a) BMD alone is insufficient for fracture risk stratification in PLWH, as more than one-quarter of patients with normal BMD had degraded microarchitecture; (b) simple bedside functional tests (grip strength and gait speed) can serve as rapid screening tools to identify patients with compounded skeletal vulnerability; and (c) patients with prolonged TDF exposure (>5 years) should be prioritized for comprehensive bone quality evaluation, including TBS and/or calcaneal QUS, even when standard densitometry appears reassuring.

From a resource-utilization perspective, the multimodal screening pathway proposed in this study is designed to be cost-efficient and feasible in resource-limited settings. The sarcopenia screening tools—handgrip dynamometry and 4-m gait speed—require minimal equipment investment (less than €50 one-time cost) and add fewer than 5 min to a routine clinic visit. Calcaneal QUS devices are portable and substantially less expensive than DXA scanners, making them suitable triage tools in settings where densitometry access is constrained. TBS analysis is derived from existing lumbar DXA images using dedicated software, requiring no additional radiation exposure or scan time. Thus, the proposed approach layers inexpensive functional and ultrasound-based assessments onto existing DXA-based workflows, potentially improving fracture risk stratification at minimal incremental cost. Future prospective studies incorporating formal cost-effectiveness analyses would help quantify the health-economic value of this integrated screening strategy.

### 4.2. Study Limitations

The cross-sectional, single-center design limits causal inference and generalizability, and referral-based recruitment for bone evaluation may enrich higher-risk profiles (selection bias). The sarcopenia assessment was a pragmatic screen (grip and gait) rather than a full consensus diagnostic framework including muscle mass imaging, which may misclassify some participants. Fracture analyses were underpowered (low event counts), and residual confounding (lifetime ART details, endocrine factors, nutrition, physical activity) may persist despite adjustment, particularly for complex relationships between turnover, ART exposure, and microarchitecture. The contribution of boosted protease inhibitor exposure to bone outcomes was not separately analyzed due to the limited number of patients on such regimens and the cross-sectional design, which precluded detailed ART-stratified subanalyses.

## 5. Conclusions

Among PLWH, sarcopenia screen-positive status and prolonged TDF exposure were associated with worse bone microarchitecture and complementary bone quality signals (lower TBS and QUS SOS) alongside higher turnover. Degraded TBS was markedly more prevalent with >5 years TDF exposure, while viral suppression emerged as an independent protective correlate of microarchitectural integrity. Together, the results favor integrating TBS, QUS, and brief functional testing into feasible, risk-stratified bone health pathways for PLWH, especially where densitometry access is constrained.

## Figures and Tables

**Table 1:** Baseline characteristics by sarcopenia screen status.

Variable	No Sarcopenia (*n* = 70)	Sarcopenia (*n* = 28)	*p*-Value
Age, years	42.8 ± 7.6	53.4 ± 7.7	<0.001
Female sex, n (%)	20 (28.6)	10 (35.7)	0.486
BMI, kg/m^2^	25.2 ± 3.1	23.9 ± 3.8	0.094
Current smoker, n (%)	22 (31.4)	11 (39.3)	0.458
HIV duration, years	10.3 ± 4.5	12.6 ± 5.2	0.03
Nadir CD4+, cells/mm^3^	258.7 ± 105.9	206.3 ± 107.2	0.029
Current CD4+, cells/mm^3^	578.0 ± 176.3	534.4 ± 173.0	0.294
Viral suppression < 50 copies/mL, n (%)	60 (85.7)	17 (60.7)	0.006
Cumulative TDF exposure, years	4.1 ± 3.3	5.4 ± 3.7	0.11
25(OH) vitamin D, ng/mL	24.0 ± 8.0	20.7 ± 7.7	0.062
CTX, ng/mL	0.4 ± 0.2	0.6 ± 0.2	<0.001
Grip strength, kg	33.7 ± 7.1	23.1 ± 4.1	<0.001
Gait speed, m/s	1.1 ± 0.2	0.7 ± 0.1	<0.001
Lumbar spine T-score	−1.2 ± 0.9	−1.7 ± 0.9	0.014
Total hip T-score	−0.9 ± 0.8	−1.2 ± 0.8	0.095
Trabecular bone score (TBS × 100)	127.7 ± 6.8	123.8 ± 7.0	0.02
Calcaneal SOS, m/s	1548.8 ± 37.1	1523.3 ± 35.0	0.002
Morphometric vertebral fracture, n (%)	8 (11.4)	7 (25.0)	0.086

BMI, body mass index; CD4+, cluster of differentiation 4 positive T lymphocytes; CTX, C-terminal telopeptide of type I collagen; HIV, human immunodeficiency virus; n, number; SOS, speed of sound; TBS, trabecular bone score; TDF, tenofovir disoproxil fumarate.

**Table 2:** Bone and biomarker profiles by cumulative TDF exposure.

Variable	Never (*n* = 30)	≤5y (*n* = 40)	>5y (*n* = 28)	*p*-Value
Age, years	42.3 ± 8.3	46.1 ± 9.3	49.3 ± 8.1	0.004
BMI, kg/m^2^	25.5 ± 3.0	24.8 ± 3.4	23.9 ± 3.8	0.108
Lumbar spine T-score	−0.9 ± 0.8	−1.2 ± 0.9	−1.7 ± 0.9	<0.001
Total hip T-score	−0.6 ± 0.7	−0.9 ± 0.8	−1.3 ± 0.8	<0.001
Femoral neck T-score	−0.9 ± 0.7	−1.2 ± 0.8	−1.6 ± 0.8	<0.001
Trabecular bone score (TBS × 100)	129.8 ± 6.3	127.0 ± 6.6	123.1 ± 7.1	<0.001
Calcaneal SOS, m/s	1557.7 ± 33.9	1545.0 ± 37.0	1524.2 ± 36.8	<0.001
Calcaneal BUA, dB/MHz	111.6 ± 10.8	109.4 ± 12.1	104.8 ± 13.8	0.034
25(OH) vitamin D, ng/mL	24.6 ± 8.2	23.1 ± 7.8	20.6 ± 7.6	0.113
CTX, ng/mL	0.4 ± 0.2	0.4 ± 0.2	0.6 ± 0.2	<0.001
P1NP, µg/L	48.8 ± 16.8	51.7 ± 18.0	60.0 ± 20.1	0.02
Degraded TBS (<124.0), n (%)	12 (40.0)	19 (47.5)	23 (82.1)	0.001
Morphometric vertebral fracture, n (%)	3 (10.0)	6 (15.0)	6 (21.4)	0.423

BMI, body mass index; BUA, broadband ultrasound attenuation; CTX, C-terminal telopeptide of type I collagen; *n*, number; P1NP, procollagen type 1 N-terminal propeptide; SOS, speed of sound; TBS, trabecular bone score; TDF, tenofovir disoproxil fumarate; 25(OH) vitamin D, 25-hydroxyvitamin D.

**Table 3:** Joint BMD–TBS phenotypes and vertebral fracture prevalence.

BMD Category	n	TBS Normal n (%)	TBS Partially Degraded n (%)	TBS Degraded n (%)	Vertebral Fracture n (%)
Normal	37	18 (48.6)	9 (24.3)	10 (27.0)	3 (8.1)
Osteopenia	44	10 (22.7)	17 (38.6)	17 (38.6)	8 (18.2)
Osteoporosis	17	0 (0.0)	7 (41.2)	10 (58.8)	4 (23.5)

TBS distribution across BMD categories (chi-square) *p* = 0.006; vertebral fracture prevalence across BMD categories *p* = 0.236. BMD, bone mineral density; n, number; TBS, trabecular bone score.

**Table 4:** Correlations between TBS × 100 and clinical/biochemical variables.

Predictor	Pearson r	*P*-Value
Age, years	−0.4	<0.001
BMI, kg/m^2^	0.2	0.061
HIV duration, years	−0.3	0.002
Nadir CD4+, cells/mm^3^	0.1	0.367
Lumbar spine T-score	0.6	<0.001
Total hip T-score	0.5	<0.001
Calcaneal SOS, m/s	0.4	<0.001
CTX, ng/mL	−0.3	0.001
25(OH) vitamin D, ng/mL	0.2	0.045
Grip strength, kg	0.4	<0.001
Gait speed, m/s	0.4	<0.001

BMI, body mass index; CTX, C-terminal telopeptide of type I collagen; HIV, human immunodeficiency virus; r, Pearson correlation coefficient; SOS, speed of sound; TBS, trabecular bone score; 25(OH) vitamin D, 25-hydroxyvitamin D.

**Table 5:** Multivariable logistic regression for degraded TBS (TBS × 100 < 124.0).

Predictor	aOR	95% CI	*p*-Value
Age (per 10 years)	1.4	0.9–2.0	0.106
BMI (per 1 kg/m^2^)	0.9	0.8–1.0	0.072
HIV duration (per 5 years)	1.1	0.8–1.4	0.614
Nadir CD4+ < 200 (yes vs. no)	1.4	0.6–3.4	0.466
TDF exposure > 5y (yes vs. no)	1.4	0.5–3.5	0.517
Viral suppression present (yes vs. no)	0.3	0.1–0.9	0.034
Sarcopenia (yes vs. no)	1.6	0.6–4.3	0.343
CTX (per 0.1 ng/mL)	1.1	0.9–1.4	0.274

aOR, adjusted odds ratio; BMI, body mass index; CI, confidence interval; CTX, C-terminal telopeptide of type I collagen; HIV, human immunodeficiency virus; TBS, trabecular bone score; TDF, tenofovir disoproxil fumarate.

**Table 6:** Sequential discrimination models for prevalent vertebral fractures.

Model	Variables Included	AUC × 100	ΔAUC × 100 vs. Prior	LRT *p*-Value vs. Prior
A	Clinical: age, sex, BMI, smoking, HIV duration, nadir CD4+ < 200, cumulative TDF years	68.3		
B	Model A + lowest T-score (lumbar spine/hip/femoral neck)	72.5	4.2	0.081
C	Model B + TBS × 100	74	1.5	0.484
D	Model C + sarcopenia	75.6	1.6	0.377

AUC, area under the receiver operating characteristic curve; BMI, body mass index; HIV, human immunodeficiency virus; LRT, likelihood ratio test; TBS, trabecular bone score; TDF, tenofovir disoproxil fumarate.

**Table 7:** Mean vs. lower-tail determinants of TBS.

Predictor	OLS (Mean) β (95% CI)	OLS (Mean) p	Quantile τ = 0.25 β (95% CI)	Quantile τ = 0.25 p
Probable sarcopenia (yes vs. no)	−2.3 (−6.1 to 1.5)	0.238	−0.0 (−4.4 to 4.3)	0.993
CTX (ng/mL)	−2.2 (−9.4 to 5.1)	0.553	−4.1 (−13.7 to 5.4)	0.391
Cumulative TDF exposure (years)	−0.5 (−0.8 to −0.1)	0.018	−0.6 (−1.1 to −0.1)	0.023
25(OH) vitamin D (ng/mL)	−0.0 (−0.2 to 0.1)	0.635	−0.0 (−0.2 to 0.2)	0.836
Age (years)	−0.2 (−0.4 to −0.0)	0.013	−0.3 (−0.5 to −0.0)	0.034
BMI (kg/m^2^)	0.1 (−0.3 to 0.5)	0.655	0.3 (−0.2 to 0.8)	0.297
Viral suppression (yes vs. no)	−0.1 (−3.7 to 3.4)	0.938	−0.6 (−5.1 to 3.9)	0.782

BMI, body mass index; CI, confidence interval; CTX, C-terminal telopeptide of type I collagen; OLS, ordinary least squares; TBS, trabecular bone score; TDF, tenofovir disoproxil fumarate; τ, quantile level; 25(OH) vitamin D, 25-hydroxyvitamin D.

**Table 8:** Partial-correlation network controlling for age, BMI, and HIV duration.

Var 1	Var 2	Partial r (×100)	*p*	q (BH-FDR)
TBS (×100)	Calcaneal SOS (m/s)	61.1	<0.001	<0.001
Lumbar spine T-score	Calcaneal SOS (m/s)	38.2	<0.001	0.001
TBS (×100)	Lumbar spine T-score	35.9	<0.001	0.002
Gait speed (m/s)	25(OH) vitamin D (ng/mL)	−20.2	0.046	0.242
Grip strength (kg)	Gait speed (m/s)	17.1	0.091	0.384
TBS (×100)	CTX (ng/mL)	−14.7	0.148	0.517
CTX (ng/mL)	25(OH) vitamin D (ng/mL)	−13	0.203	0.608
Calcaneal SOS (m/s)	Grip strength (kg)	−11.8	0.246	0.647
Calcaneal SOS (m/s)	25(OH) vitamin D (ng/mL)	10.4	0.306	0.714
TBS (×100)	Grip strength (kg)	9.1	0.374	0.785
Calcaneal SOS (m/s)	CTX (ng/mL)	−6.8	0.504	0.963
Lumbar spine T-score	Grip strength (kg)	−6.3	0.54	0.945
CTX (ng/mL)	Grip strength (kg)	−4.3	0.672	1
TBS (×100)	25(OH) vitamin D (ng/mL)	4	0.693	1
Grip strength (kg)	25(OH) vitamin D (ng/mL)	−4	0.695	0.972
Lumbar spine T-score	CTX (ng/mL)	−3.3	0.745	0.978
CTX (ng/mL)	Gait speed (m/s)	−3	0.773	0.955
Calcaneal SOS (m/s)	Gait speed (m/s)	2.7	0.795	0.928
TBS (×100)	Gait speed (m/s)	1.4	0.891	0.985
Lumbar spine T-score	25(OH) vitamin D (ng/mL)	0.9	0.93	0.977
Lumbar spine T-score	Gait speed (m/s)	−0.5	0.964	0.964

BH-FDR, Benjamini–Hochberg false discovery rate; BMI, body mass index; CTX, C-terminal telopeptide of type I collagen; HIV, human immunodeficiency virus; *p*, *p*-value; partial r, partial correlation coefficient; q, FDR-adjusted *p*-value; SOS, speed of sound; TBS, trabecular bone score; 25(OH) vitamin D, 25-hydroxyvitamin D.

## Data Availability

The data presented in this study are available on request from the corresponding author.
